# Cross-Protective Peptide Vaccine against Influenza A Viruses Developed in HLA-A*2402 Human Immunity Model

**DOI:** 10.1371/journal.pone.0024626

**Published:** 2011-09-19

**Authors:** Toru Ichihashi, Reiko Yoshida, Chihiro Sugimoto, Ayato Takada, Kiichi Kajino

**Affiliations:** 1 Department of Collaboration and Education, Hokkaido University Research Center for Zoonosis Control, Sapporo, Japan; 2 Department of Global Epidemiology, Hokkaido University Research Center for Zoonosis Control, Sapporo, Japan; Instituto Butantan, Brazil

## Abstract

**Background:**

The virus-specific cytotoxic T lymphocyte (CTL) induction is an important target for the development of a broadly protective human influenza vaccine, since most CTL epitopes are found on internal viral proteins and relatively conserved. In this study, the possibility of developing a strain/subtype-independent human influenza vaccine was explored by taking a bioinformatics approach to establish an immunogenic HLA-A24 restricted CTL epitope screening system in HLA-transgenic mice.

**Methodology/Principal Findings:**

HLA-A24 restricted CTL epitope peptides derived from internal proteins of the H5N1 highly pathogenic avian influenza A virus were predicted by CTL epitope peptide prediction programs. Of 35 predicted peptides, six peptides exhibited remarkable cytotoxic activity *in vivo*. More than half of the mice which were subcutaneously vaccinated with the three most immunogenic and highly conserved epitopes among three different influenza A virus subtypes (H1N1, H3N2 and H5N1) survived lethal influenza virus challenge during both effector and memory CTL phases. Furthermore, mice that were intranasally vaccinated with these peptides remained free of clinical signs after lethal virus challenge during the effector phase.

**Conclusions/Significance:**

This CTL epitope peptide selection system can be used as an effective tool for the development of a cross-protective human influenza vaccine. Furthermore this vaccine strategy can be applicable to the development of all intracellular pathogens vaccines to induce epitope-specific CTL that effectively eliminate infected cells.

## Introduction

Influenza A viruses are highly contagious and cause respiratory tract infection associated with a marked disease burden. Inactivated influenza A virus vaccines that stimulate the production of antibodies to surface glycoproteins hemagglutinin (HA) and neuraminidase (NA) are currently available. The protective role of antibodies against HA in particular is well established and has been demonstrated in both infected animals and humans [1]. While the induction of neutralizing antibody production against a particular viral strain with a matching inactivated vaccine is generally effective for reducing the intensity of clinical -signs [2], the effects of these vaccines are limited due to their inability to stimulate mucosal immunity and cytotoxic T lymphocyte (CTL) responses, both of which are indispensable for the suppression of initial viral replication in the respiratory epithelium [3], [4]. Furthermore, antibodies induced by inactivated vaccines fail to protect against infection with different influenza A virus subtypes or homologous virus strains due to the effect of antigenic drift at the neutralizing antibody combining site [5].

In contrast to inactivated vaccines, live attenuated influenza vaccines (LAIV) which are currently licensed in the United States are administered intranasally (i.n.) and induce cross-protective immunity by stimulating mucosal immunity and CTL responses. Clinical studies indicate that LAIV provide increased protection against seasonal influenza compared to inactivated vaccines, especially in young children, even when vaccine strains are sub-optimally matched to circulating strains [6]. However another report has suggested that LAIV are not as effective as generally thought, because protective effect may vary by age and population [7].

Recently, it has been reported that, compared to formalin or UV inactivated vaccine, gamma irradiated influenza virus provide significant cross-protection against several virus strains including highly pathogenic H5N1 subtype [8]. Although LAIV and gamma irradiated vaccine facilitate cross-protection, the underlying mechanism of cross-protective immunity and the role of CTLs in mediating cross-protective immunity remain important areas of research.

CTLs have been shown to play a significant role in the control of primary influenza A virus infection in mice [9], [10]. Since most CTL epitopes that are found on internal viral proteins are relatively conserved, CTLs induced by primary infection are able to contribute to protective immunity against influenza viruses of various subtypes [11]–[14]. The protective effect mediated by CTLs has been confirmed by the adoptive transfer of virus-specific CTLs that show a protective effect during the course of infection in mice [15]–[18] and also by the depletion of CTLs from infected mice which lead to a more severe disease state and increased mortality associated with rapid virus replication in the lung [19]. Thus, cross-protective immunity is mediated at least in part by CTLs that recognize conserved influenza A virus epitopes [20]–[22].

Synthetic peptides are the most desirable material for epitope specific CTL inducing vaccines since they are relatively easy to produce [23]. Because peptides can be synthesized artificially, stored lyophilized at room temperature and easily modified with chemical compounds. However, peptide vaccines have several disadvantages including the limitations conferred by major histocompatibility complex (MHC) restriction and poor immunogenicity. Firstly, CTLs recognize peptide epitopes presented in a complex with class I MHC molecules, therefore peptides require substantial affinity for MHC. Polymorphisms of human leukocyte antigen (HLA, human MHC) add additional complexity since peptide epitopes need to be designed with respect to each HLA haplotype. Several web based prediction programs with modified algorithms have been designed to predict MHC binding peptides that are able to cover several major HLA supertypes, thereby making it possible to narrow down the candidate epitopes which are able to bind to specific HLA haplotypes [24]–[27]. HLA-A*2402 is one of the major HLA supertypes with approximately 60% of the population in Japan being positive for this specific HLA subtype [28]. Despite being commonly expressed, little is known about its ability to bind peptides derived from influenza A virus. Secondly, because CTL responses are difficult to induce with epitope peptides alone, the addition of an adjuvant or chemical modification is required for the creation of an effective peptide vaccine. For example, the addition of potent immune-enhancing adjuvants such as Poly (I∶C) or the toll-like receptor (TLR) agonist CpG-ODN facilitates the induction of sufficient CTL responses [29]. In addition, peptide immunogenicity can be enhanced by cross-linking the N- or C-terminus of the peptide with carrier molecules such as liposomes. These modifications allow the delivery of peptide antigens to professional antigen presenting cells for subsequent enhancement of T cell responses [30]. Thus, the combination of a carrier conjugated 8–10 mer peptide and a nucleic acid based adjuvant, such as CpG-ODN, could make an ideal CTL inducing vaccine.

We reported previously that a liposome conjugated murine class I MHC restricted NP_366–374_ peptide epitope derived from the influenza strain A/Aichi inhibited virus replication in the lung [29]. Here, we have established a HLA-A*2402 restricted CTL activating peptide selection system using HLA binding peptide prediction programs and HLA-A24 transgenic mice to develop a human influenza peptide vaccine. Peptide epitopes of highly pathogenic avian influenza A virus proteins were screened and peptides that induced CTL activity were subjected to a protection test against different influenza A virus subtypes using a human CTL immunity mouse model.

## Materials and Methods

### Viruses

Influenza A virus strains A/HK483 (A/HongKong/483/97 [H5N1]), A/PR8 (A/PuertoRico/8/34 [H1N1]) and A/Aichi (A/Aichi/2/68/ [H3N2]) were propagated in the allantoic cavity of 10- to 11-day-old embryonated hen's eggs at 35°C for 48 h. The amniotic/allantoic fluids were harvested, pooled, and stored at −80°C. Titers were determined using plaque assays on Madin-Darby canine kidney (MDCK) cells, as described previously [31]. The viruses were kindly provided by Prof. Hiroshi Kida from Laboratory of Microbiology, Graduate School of Veterinary Medicine, Hokkaido University (Sapporo, Japan).

### Peptides

The 9 mer peptides corresponding to the HLA-A*2402 binders were predicted by the web-based programs: BIMAS (http://www-bimas.cit.nih.gov/molbio/hla_bind/), nHLAPred (http://www.imtech.res.in/raghava/nhlapred/neural.html), SYFPEITHI (http://www.syfpeithi.de/Scripts/MHCServer.dll/EpitopePrediction.htm) and NetCTL (http://www.cbs.dtu.dk/services/NetCTL/). Predicted putative CTL epitope peptides derived from internal proteins of influenza virus A/HK483 were manufactured, HPLC purified (>90% purity), and analyzed by mass spectrometry (Invitrogen, Carlsbad, CA). The peptides were dissolved in dH_2_O at 1.0 mg/ml with or without various concentrations of NaHCO_3_ and stored at −20°C. For carrier-conjugated peptide experiments, liposomes consisting of dioleoyl phosphatidyl choline, dioleoyl phosphatidyl ethanolamine, dioleoyl phosphatidyl glycerol acid, and cholesterol in a 4∶3∶2∶7 molar ratio were provided by Nippon Oil and Fat Corporation (Tokyo, Japan) and used, as described previously [32]. The crude liposome solution was passed through a membrane filter (Nucleopore polycarbonate filter, Corning Coster) with a pore size of 0.2 µm. Liposome conjugated peptides were prepared using disuccinimidyl suberate (DSS) for cross-linking, as described previously [33]. These peptide-conjugated liposomes were kindly prepared by Dr. Uchida at Department of Safety Research on Blood and Biological Products, National Institute of Infectious Disease, Tokyo, Japan.

### Mice

HLA-A24 transgenic (A24Tg) mice (kindly provided by Dr. François Lemonnier, Département d'Immunologie, Institut Pasteur, Paris, France) were bred under specific-pathogen-free conditions. These mice have a C57BL/6 background and express HLA-A*2402, human ß2 microglobulin and CD8 molecules, but do not express either murine H2D^b^ nor H2K^b^. All experimental procedures were approved by Hokkaido University Animal Care and Use Committee (approval number 10-0060), Sapporo, Japan.

### *In vivo* cytotoxicity assays

Eight to 12 week-old A24Tg mice were immunized subcutaneously (s.c.) twice with each liposome-conjugated peptide in the presence of CpG-ODN (CpG5002, 5 µg/mouse) (Hokkaido System Science, Sapporo, Japan) or poly(I∶C) (10 µg/mouse) (InvivoGen, San Diego, CA). Splenocytes from A24Tg mice were suspended in PBS and then labeled with two different concentrations (5 µM or 0.5 µM) of carboxyfluorescein diacetate succinimidyl ester (CFDA-SE, Invitrogen) at room temperature for 10 min. After addition of equal volumes of heat inactivated rabbit serum to quench the CFSE labeling reaction, cells were washed twice with PBS. Cells were further incubated with 0.5 µM immunizing peptide or an irrelevant peptide for 2 h at 37°C and 5% CO_2_. Five million cells cultured with respective peptides were mixed together and inoculated intravenously (i.v.) into immunized mice. Eighteen hours after target cells were inoculated, splenocytes were harvested and ten thousand CFSE-positive cells were analyzed by flow cytometry with dead cell exclusion performed by propidium iodide staining (PI, Invitrogen). Peptide specific cell reduction ratios were calculated using the following formula:

ITCR (inoculated target cell ratio) = (number of immunized peptide pulsed cells harvested from PBS injected mice)/(number of irrelevant peptide pulsed cells harvested from PBS injected mice), % specific reduction = {(number of irrelevant peptide pulsed cells harvested from immunized mice)×ITCR−(number of immunized peptide pulsed cells harvested from immunized mice)}/{(number of irrelevant peptide pulsed cells harvested from immunized mice)×ITCR}×100.

### Titration of virus

MDCK cells were maintained in Dulbecco's modified Eagle's medium (DMEM) supplemented with 10% fetal bovine serum (FBS) at 37°C in a humidified atmosphere with 5% CO_2_. Suspensions of the lung cells serially diluted 1 in 10 were inoculated into confluent MDCK cell monolayers on 6-well plates and incubated at 35°C. After 1 h adsorption, the inoculum was removed and cells were overlaid with minimal essential medium (MEM) containing 1% bacto agar (BD Diagnostic Systems, Sparks, MD) and 5 µg/ml of trypsin (Invitrogen). After incubation at 35°C for 2 days in 5% CO_2_, the plaques were counted. The limit of detection in this assay was 1×10^3^ PFU/g [34].

### Virus protection tests

Eight to 12 week-old A24Tg mice were immunized s.c. or i.n. with a mixture of peptide-liposome conjugates and CpG-ODN (CpG5002, 5 µg/mouse) or poly(I∶C) (10 µg/mouse) and were anesthetized with sodium pentobarbital. The mice were re-immunized one and two weeks later. Control mice were given PBS or CpG-ODN under the same conditions. One week after the final immunization, 3 to 10 mice in each group were challenged i.n. with 50 µl of 20×50% mouse lethal doses (MLD50) of A/HK483, A/PR8 or 20×50% mouse infectious doses (MID50) of A/Aichi under anesthesia. Five days after the virus challenge, 4 to 5 mice were sacrificed to obtain tissue samples. Body weights were observed every day for 14 days after the challenge. All mice were sacrificed when a body weight loss percentage of over 25% was reached. The MLD50 and MID50 were determined by infecting 5 mice i.n. with 10 µl of serial 10-fold dilutions of the viruses.

### Immunohistochemistry

Eight to 12 week-old A24Tg mice were vaccinated i.n. with 45 µl of peptide-liposome conjugates in the presence of CpG-ODN weekly over a period of three weeks. Lungs were harvested at 7 days after the final immunization, embedded in O.C.T. compound (Sakura) and slowly frozen in dry ice-2-propanol. Ten µm thick frozen sections were prepared in a cryostat and air-dried for 1 hour at room temperature. The sections were post-fixed in acetone∶ethanol (1∶1) solution, rehydrated in PBS and incubated in excess avidin followed by incubation with excess biotin (Avidin/Biotin Blocking Kit; Vector Laboratories). The sections were stained with biotinylated hamster anti-mouse CD3 (eBioscience, San Diego, CA) or biotinylated rat anti-mouse CD8α (R&D Systems ) antibodies. Immunohistochemical staining was performed using a HistoMouse-Plus Kit (Invitrogen), according to the manufacturer's instructions.

### Statistical analyses

Statistical analyses were carried out using Student's t-test. P values<0.05 were considered significant.

## Results

### Prediction of HLA-A24 binding epitopes derived from internal proteins of influenza A virus

While the selective pressure from neutralizing antibodies induces a high frequency of antigenic drift in influenza A virus surface proteins, HA and NA , the amino acid sequences of internal proteins, such as matrix (M), nonstructural (NS), nucleocapsid (NP), polymerase acidic (PA), polymerase basic 1(PB1) and polymerase basic 2 (PB2), are relatively well-conserved. To develop a viral subtype-independent vaccine, eight internal proteins of the influenza A/HK483 strain, including two post-translational products of both the M and NS genes, were used as templates for the CTL epitope. A total of 35 epitope candidates that indicated high scores from the data processed by the HLA-A24 binding peptide prediction programs, with or without several options, were initially chosen. All predicted epitope peptides were synthesized, purified (>90% purity) and used in further experiments.

### *In vivo* validation of immunogenic CTL epitopes using A24Tg mice

To confirm whether the predicted peptides have sufficient immunogenicity to induce peptide antigen specific CTL activation, HLA-A24Tg mice were immunized with liposome-conjugated peptide weekly over two weeks. *In vivo* cytotoxicity analysis showed that 10 peptides induced CTL activity (Table 1).

**Table 1:** Screened immunogenic CTL epitopes by *in vivo* cytotoxicity assay using A24Tg mice.

Peptide	A/HK/483/97(H5N1)	A/PR/8/34(H1N1)	A/Aichi/2/34(H3N2)	%Killing^b^
PA_45–53_	CFMYSDFHF	CFMYSDFHF	CFMYSDFHF	59%
PA_130–138_	YYLEKANKI	YYLEKANKI	YYLEKANKI	95%
PB1_216–224_	SYLIRALTL	SYLIRALTL	GYLIRALTL	22%
PB1_430–438_	RYTKTTYWW	RYTKTTYWW	KYTKTTYWW	95%
PB1_482–490_	SYINRTGTF	SYINRTGTF	SYINKTGTF	56%
PB1_688–696_	MYQKCCTLF	MYQRCCNLF	MYQKCCNLF	89%
PB2_117–125_	TYFEKVERL	TYFERVERL	TYFDKVERL	13%
PB2_322–330_	SFSFGGFTF	SFSFGGFTF	SFSFGGFTF	38%
PB2_549–557_	TYQWIIRNW	TYQWIIRNW	TYQWVIRNW	95%
M2_40–48_	LWILDRLFF	LWILDRLFF	LWILDRLFF	5%
Tyrosinase_206–214_ a	AFLPWHRLF			96%

a HLA-A*2402 binding high immunogenic peptide, which is unrelated to influenza virus antigen.

b *In viv*o peptide specific cell reduction ratios were calculated using the formula described in Materials and Methods.

Among the six peptides that showed high immunogenicity (more than 50% killing), five peptides were identical between A/HK483 and A/PR8, and two of these were also identical among the three viral subtypes including A/Aichi.

To demonstrate the restriction of these peptides to HLA-A*2402, C57BL/6 mice which were background of A24Tg mice were immunized with three highly immunogenic HLA-A*2402 restricted peptides and *in vivo* cytotoxicity assay was performed. This result showed that the immunogenicity of the peptides were not provoked by strain background but by transgenic HLA-A*2402 (Figure S1). Furthermore, we performed HLA stabilization assay using RMA-S cells expressing HLA-A*2402 (RMA-S- A*2402 cells) to demonstrate the restriction of the peptides to HLA-A*2402. The surface expression of HLA-A*2402 on RMA-S- A*2402 cells was stabilized in a dose-dependent manner when cells were cultured with three highly immunogenic HLA-A*2402 restricted peptides, whereas the expression was not stabilized when cells were cultured with irrelevant peptide (Figure S2). Therefore, the restrictions of highly immunogenic peptides to HLA-A*2402 were confirmed.

### Single epitope vaccinated mice exhibited limited protection against H5N1 virus infection

**Figure 1:**
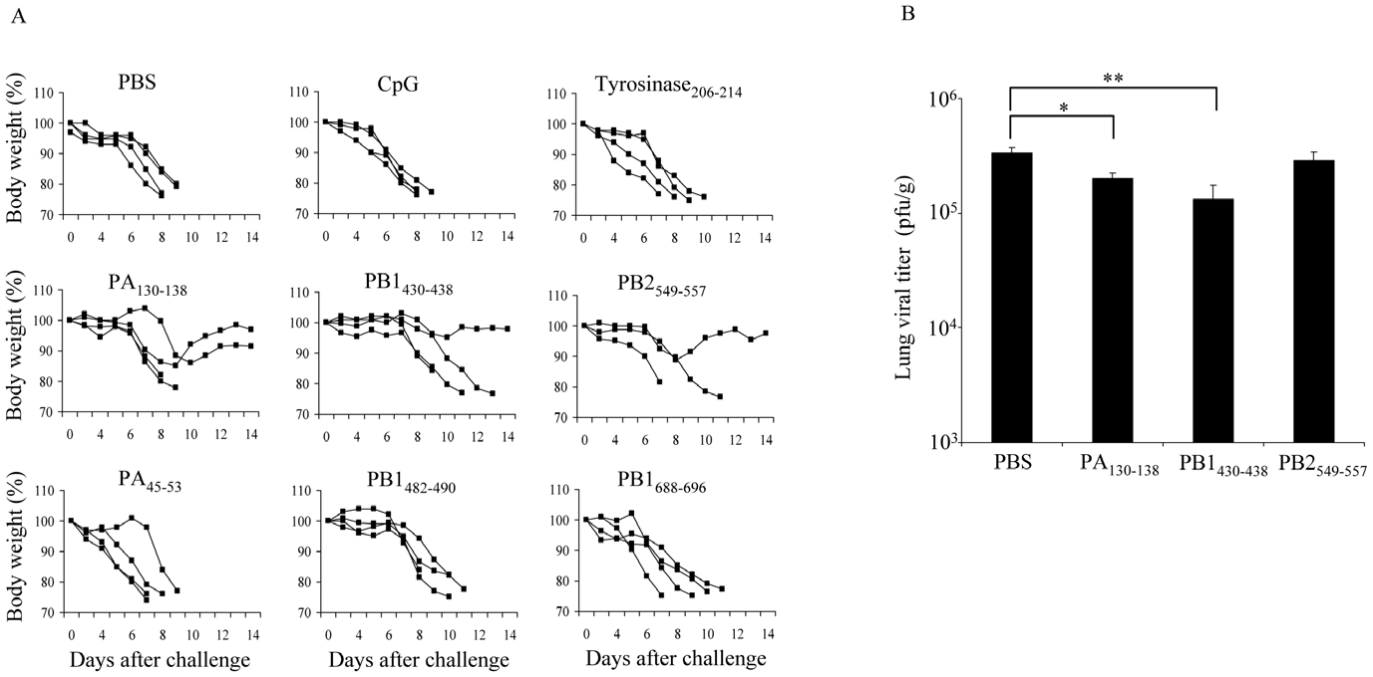
Protective effect of single epitope vaccinated mice is not sufficient against lethal virus challenge. A24Tg mice were immunized i.n. three times at 7 to 9 days intervals with each peptide in the presence of CpG-ODN, PBS alone or CpG-ODN plus empty-liposome solution, and then infected i.n. with lethal dose of A/HK483 (H5N1) (40 PFU/mouse) 1 week after the final immunization. The survival and the body weight were monitored for 14 days (A). On day 5 post-infection, lung viral titers of PA_130–138_, PB1_430–438_ or PB2_549–557_ peptide immunized mice were determined by calculating TCID_50_ using MDCK cells as described in Materials and Methods (B). *p<0.05. , **p<0.01.

In order to elucidate the potency of each of the six highly immunogenic peptides, we examined the protective effect of each individual peptide against influenza A/HK483 virus infection. The A24Tg mice were immunized i.n. with each peptide three times at 7 to 9 day intervals and were then infected with A/HK483 virus 1 week after the final immunization. Although unimmunized mice began losing body weight at day 4, mice immunized with PA_130–138_, PB1_430–438_ or PB2_549–557_ began losing weight at day 7 or later. Moreover, 25 to 50% of mice that had been immunized with PA_130–138_, PB1_430–438_ or PB2_549–557_ survived after exposure to a lethal dose of virus and exhibited milder clinical signs (Figure 1A). To exclude the possibility that non-specific protection of the irrelevant peptide or CpG-ODN diminished infectivity, mice were also immunized i.n. with Tyrosinase_206–214_ or CpG-ODN plus empty-liposome solution before being challenged with a lethal dose of A/HK483 virus. In the result, non-specific protection by Tyrosinase_206–214_ or CpG-ODN was not observed (Figure 1A). Moreover non-specific lung tissue disruption by CpG-ODN administration was also not observed (Figure S3).

From these results, we hypothesized that reduced mortality rate and severity of disease correlated with an inhibition of viral replication in the lung. A comparison of virus titers in the lungs of immunized and unimmunized mice 5 days after influenza challenge showed that although the titers in A24Tg mice immunized with PA_130–138_ or PB1_430–438_ decreased with statistical significance compared to that of unimmunized mice, the titers were still high to provide complete protection (Figure 1B). The correlation between immunogenicity and protection was indicated but the induction of single epitope specific CTL was not sufficient for protection against lethal influenza A virus infection.

### Nasal vaccination with multiple highly immunogenic CTL epitopes provides complete protection

To enhance the protective effect of the immunogenic peptides, three peptides (PA_130–138_, PB1_430–438_ and PB2_549–557_) from different segments of the influenza A virus genome which displayed the highest killing activity (Table 1) were chosen as peptide vaccine candidates. As a comparison, other three peptides set (PA_45–53_, PB1_482–490_, PB1_688–696_) were also chosen.

As previously reported, mucosally induced CTLs mediate effective protection against mucosal pathogens [35] in the same way i.n. administered formalin-inactivated virus vaccines effectively elicit broad spectrum humoral immunity [30]. Therefore, the vaccine efficacy of immunization route was compared.

**Figure 2:**
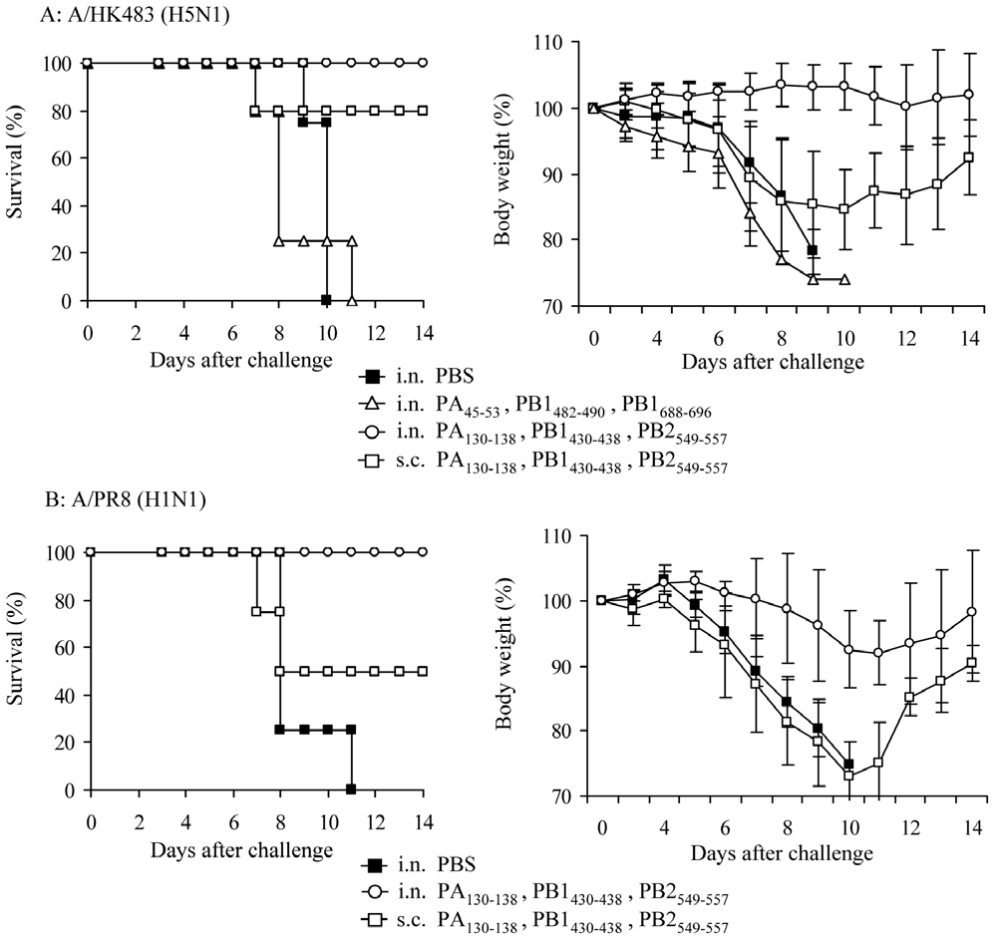
Intranasal vaccination with multiple CTL epitopes provides complete protection against lethal viral challenge. A24Tg mice were immunized i.n. or s.c. three times at 7 to 9 days intervals with the mixture of PA_130–138_, PB1_430–438_ and PB2_549–557_ peptides or the mixture of PA_45–53_, PB1_482–490_ and PB1_688–696_ peptides in the presence of CpG-ODN, unimmunized mice were administrated i.n. with PBS alone. Seven to 10 days after the final immunization, mice were challenged with A/HK483 (H5N1) (40 PFU/mouse) (A) or A/PR8 (H1N1) (1000 PFU/mouse) (B), and the survival and the body weight were monitored for 14 days.

Mice were vaccinated i.n. or s.c. three times with the mixture of three peptides, and then challenged 7 to 10 days after the final immunization with A/HK483 (40 PFU/mouse) or A/PR8 (1,000 PFU/mouse). More than half of the s.c. vaccinated mice survived after lethal infection with both viral subtypes, however they showed clinical signs similar to those of unvaccinated mice until day 8 after viral challenge. In contrast, all mice i.n. vaccinated with three peptides (PA_130–138_, PB1_430–438_ and PB2_549–557_) survived the lethal dose infection, with no body weight loss (A/HK483 infection) or diminished body weight reduction (A/PR8 infection) observed (Figure 2). On the other hand, mice vaccinated with another peptide combination (PA_45–53_, PB1_482–490_, PB1_688–696_) did not survive. This result indicated that mixing of the peptides, which did not show the protective effect by single peptide vaccination, did not provide protection against lethal virus challenge.

Thus, our data demonstrated that i.n. administration of a CTL inducing peptide vaccine provided enhanced protection compared to when s.c. immunization was performed. In addition, intranasal vaccination with multiple epitopes, which showed some survival protection effect by single peptide vaccination, induced complete protection against lethal virus challenge.

### Early viral clearance in the lung of intranasally vaccinated mice

**Figure 3:**
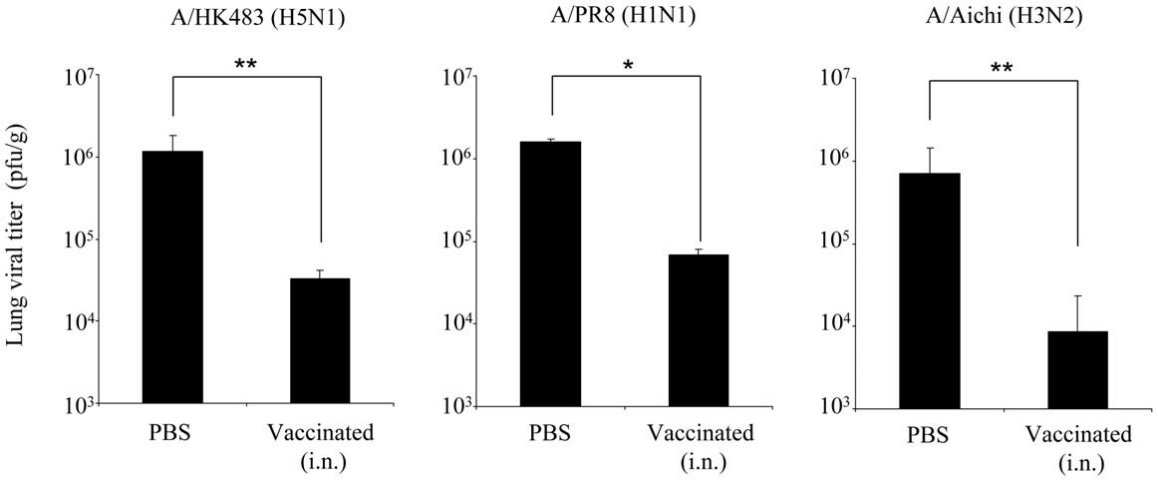
Early viral clearance can be introduced by i.n. immunization using a three peptide combination vaccine. A24Tg mice were immunized i.n. three times at 7 to 9 days intervals with the mixture of PA_130–138_, PB1_430–438_ and PB2_549–557_ peptides in the presence of CpG-ODN, or PBS alone. A week after the final immunization, mice were challenged with A/HK483 (40 PFU/mouse), A/PR8 (1000 PFU/mouse) or A/Aichi (500 PFU/mouse). On day 5 post-infection, lung viral titers were determined by calculating TCID_50_ using MDCK cells as described in Materials and Methods. *p<0.05. , **p<0.01.

Compared to the early onset of clinical signs in s.c. immunized mice, i.n. immunized mice exhibited a marked reduction in the severity of clinical signs with delayed manifestation. Therefore we speculated that i.n. vaccinated mice were able to achieve early viral clearance in the lung. Following this, i.n. immunized or unimmunized A24Tg mice were challenged with A/HK483 (40 PFU/mouse), A/PR8 (1,000 PFU/mouse) and A/Aichi (500 PFU/mouse) respectively and lung viral titers were assessed 5 days after viral challenge. Compared to the unvaccinated groups, a significant decrease in viral titer was observed in all vaccinated groups. In particular, the A/HK483 and A/Aichi virus infected groups showed a reduction of up to 2 logs average viral titers (Figure 3). These results support the notion that early viral clearance can be induced by i.n. immunization using a peptide vaccine.

### Intranasal vaccination with immunogenic peptides induces peribronchiolar recruitment of CD8^+^ T cells

**Figure 4:**
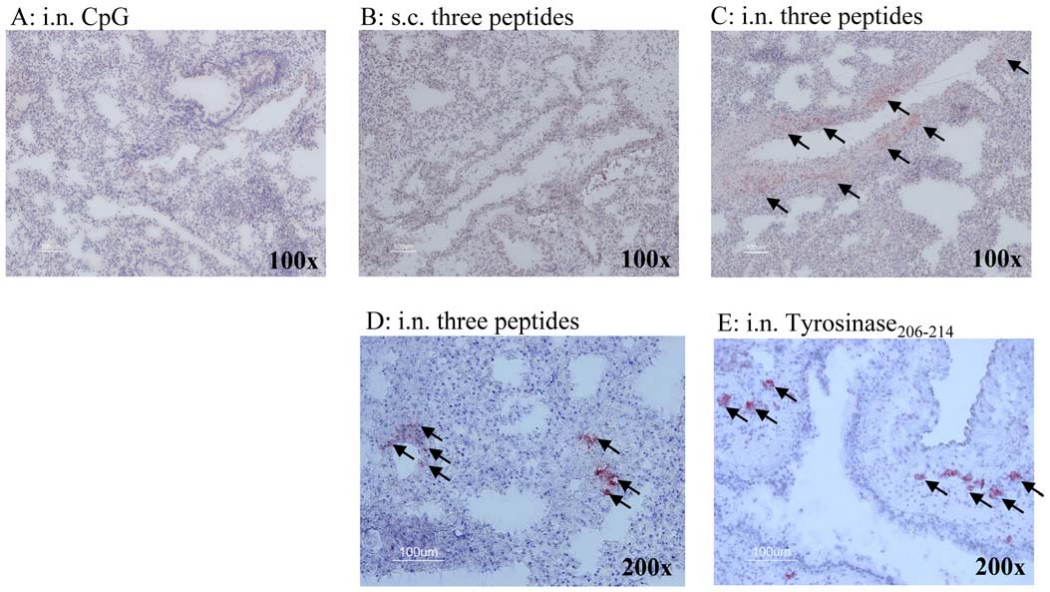
An accumulation of murine CD3^+^ and CD8^+^ cells around the bronchioles in intranasally immunized mice. A24Tg mice were immunized three times at 7 to 9 days intervals i.n.(A,C,D,E) or s.c.(B) with PA_130–138_, PB1_430–438_ and PB2_549–557_ peptides in the presence of CpG-ODN (B,C,D), Tyrosinase_206–214_ plus CpG-ODN (E) or CpG-ODN plus empty-liposome solution (A). Lungs were harvested at day 7 after the final immunization, embedded in O.C.T. compound, frozen in dry ice-2-propanol. Ten µm thick frozen sections were prepared. The sections were post-fixed in acetone∶ethanol (1∶1) solution and blocked endogenous avidin and biotin activity, then stained with anti-mouse CD3 (A,B,C) or anti-mouse CD8α (D,E).

To clarify the contribution of CTLs to early viral clearance induced by i.n. peptide vaccination, we evaluated whether or not T cells are present in lung tissue at the time point of virus infection. Mice were vaccinated i.n. or s.c. weekly for three weeks with the mixture of PA_130–138_, PB1_430–438_ and PB2_549–557_ peptides in the presence of CpG-ODN, Tyrosinase_206–214_ plus CpG-ODN or CpG-ODN plus empty-liposome solution and lungs were harvested one week after the final immunization. Lungs from mice i.n. immunized with three peptides or Tyrosinase_206–214_ showed an accumulation of murine CD3^+^ and CD8^+^ cells around the bronchioles. Moreover Gr1 positive cells as inflammatory cells were also detected in immunized mice lung (data not shown). In contrast, no CD3^+^ cell infiltration was observed in the lungs of mice s.c. immunized with three peptides or i.n. immunized with CpG-ODN plus empty-liposome solution (Figure 4). Tyrosinase_206–214_ induced CD8^+^ cells around the bronchioles (Figure 4), but all A24Tg immunized with Tyrosinase_206–214_ died after lethal A/HK483 infection (Figure 1A). This result demonstrated that the presence of effector CD8^+^ T cells induced by irrelevant peptide and the inflammatory milium they create do not provide protection against viral challenge.

Therefore our data indicates that i.n. inoculation of immunogenic peptides induced peribronchiolar T cells infiltration and the accumulation of T cells induced by immunogenic peptides derived from influenza virus in lung is required for early viral clearance of lung.

### Efficacy of long-lasting protection against lethal virus challenge in vaccinated mice

**Figure 5:**
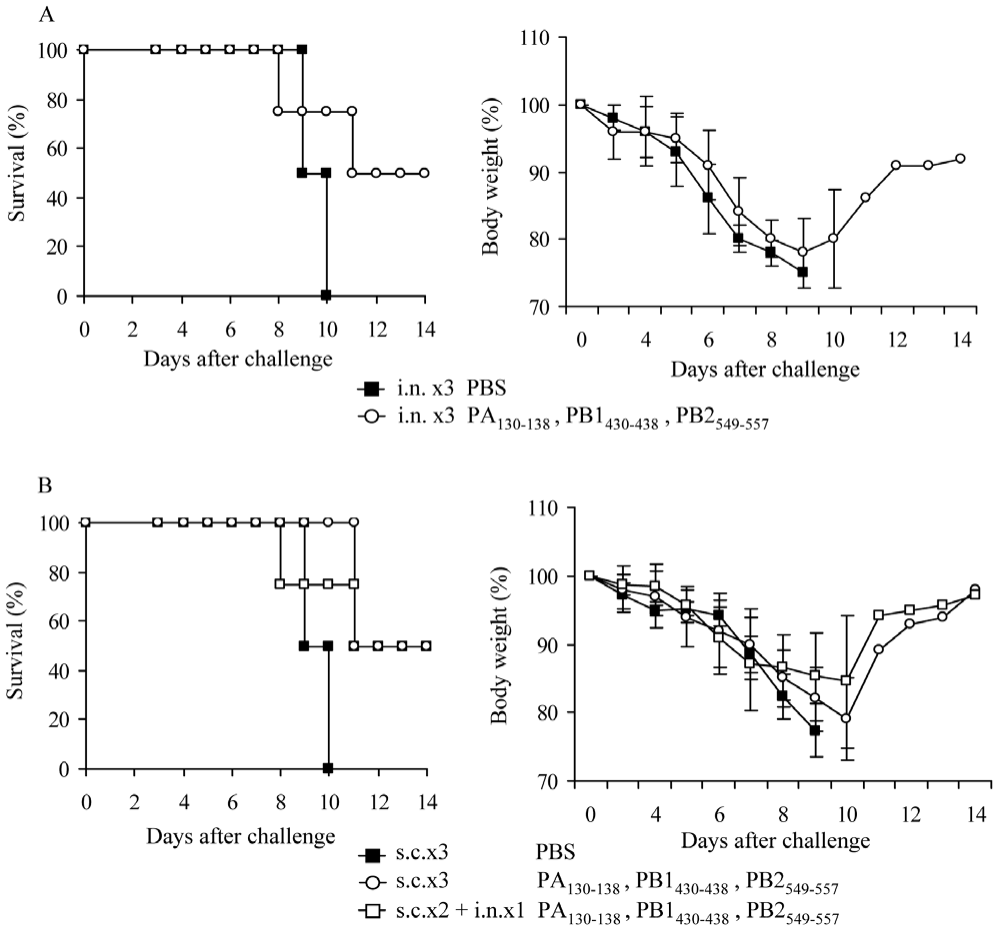
Maintenance of long-lasting protection against lethal dose challenge in vaccinated mice. A24Tg mice were immunized i.n. three times (A), s.c. three times or s.c. twice followed by i.n. (B) with PA_130–138_, PB1_430–438_ and PB2_549–557_ peptides at 7 to 9 days intervals, unimmunized mice were administrated with PBS. Eight weeks after the final immunization, mice were challenged i.n. with lethal dose of A/HK483 virus, and the survival and the body weight were monitored for 14 days.

Finally, we evaluated the efficacy of long-lasting protection induced by peptide vaccine against influenza A virus, which is a requirement for the practical use of vaccines. A24Tg mice were i.n. immunized three times with the mixture of PA_130–138_, PB1_430–438_ and PB2_549–557_ peptides and then infected with lethal dose of A/HK483 (H5N1) virus 8 weeks later (memory phase). In contrast to the full protection offered during the effector phase (1 to 2 weeks after the final immunization) after vaccination, A24Tg mice immunized i.n. developed disease indistinguishable from that of unvaccinated mice eight days after viral infection (Figure 5A). In addition, survival rates dropped by half compared to those with full protection. The rates of body weight loss were similar to those of s.c. vaccinated mice during the effector phase. Therefore, we vaccinated A24Tg mice s.c. twice with three immunogenic peptides, with additional s.c. or i.n. vaccination after the final s.c. immunization, and then compared the protective efficacy of induced memory T cells. Although the unimmunized mice did not survive, half of the mice that were s.c. immunized three times and s.c immunized twice with an additional i.n. booster survived after challenge with a lethal dose of A/HK483 virus (Figure 5B). The difference of protective efficacy by administration route was not observed in the memory CTL phase.

## Discussion

Many vaccination strategies against influenza A virus infection have demonstrated an ability to elicit virus subtype independent cross-protective immunity [36]–[38]. Among these, the use of peptide-based CTL-inducing vaccines is well established. This approach has additionally been used to induce cancer immunity in clinical trials [39], [40]. However, the immunogenicity of epitope peptides has been a long-standing problem, preventing the induction of a sufficient immune response for infected cell removal. In this study, an *in vivo* immunogenic peptide selection system was used to identify several HLA-A*2402 restricted immunogenic CTL epitopes derived from internal proteins of the H5N1 highly pathogenic influenza A strain. We have previously shown that epitope peptides are able to induce potent CTL responses after being chemically cross-linked on the surface of liposomes [29]. In this report, we investigated the effect of different administration methods on the efficacy of a CTL peptide vaccine.

Early viral clearance after influenza A virus infection is required for a significant reduction in the severity of symptoms and the prevention of lethal viral pneumonia [15], [17], [18], [41], [42]. The findings reported here are in agreement with this concept. All A24Tg mice survived after a lethal challenge of influenza virus and avoided body weight loss when viral titers in the lung decreased by approximately 100-fold after mixed peptide i.n. vaccination. Mice vaccinated with s.c. showed a delayed protective effect compared to i.n. vaccination of the same peptide. This suggests that CTLs induced by s.c. immunization do not destroy the infected airway epithelial cells immediately, although they are able to kill i.v. injected target cells effectively.

Therefore, the vaccination route of epitope peptides is one of the most important factors for facilitating protection against influenza A virus infection. The presence of CTLs that specifically recognize virally infected cells is also required in airway tissue for effective protection.

The contribution of viral antigen specific CTLs in cross-recognition against influenza A virus has been proven using mouse models [43], [44] and also in human peripheral blood mononuclear cells [45]. Broadly protective vaccines against influenza A virus infection require CTL epitope peptides to be conserved and invariable. Indeed three HLA-A*2402 restricted epitope peptides; PA_130–138_, PB1_430–438_ and PB2_549–557_, which exhibit potent CTL activity *in vivo* are highly conserved among all identified influenza A virus sequences listed in the influenza sequence database (http://www.flu.lanl.gov). Although these peptides are highly immunogenic, vaccination with each of these peptides does not show a sufficient protective effect against highly pathogenic influenza A infection. Since the abundance of viral protein expressed in infected cells is a key for determining the immuno-dominance of CTL epitopes [46], the ineffectiveness of single peptide vaccines could be due to the presence of these viral epitopes, which are abundantly expressed in infected cells. Also, because the expression levels of each polymerase protein in infected cells are not comparable to the expression levels of NP and M1 (matrix protein), it would be expected that partial activation of polymerase specific CTLs would occur, thereby preventing early viral clearance.

The ultimate goal of this vaccine is to stimulate memory CTLs that have the capacity to mediate early influenza A virus clearance. However, as demonstrated in this report, although i.n. administration of the peptide vaccine protected half of the mice from a lethal dose of A/HK483 virus, a reduction in body weight was still observed (Figure 5A). This pattern of protection is similar to that observed in s.c. vaccinated mice which were infected during the effector CTL phase. This suggests that after effector CTLs accumulate within the bronchiolar tissue, they do not remain on-site as memory CTLs after i.n. epitope peptide inoculation. This notion is consistent with a previous report showing that peripheral memory T cells of lung are recruited from the circulation [47].

Several reports indicated that helper T cell assistance is significant for augmentation of viral antigen specific memory CTL response and viral clearance [48]–[50]. Therefore, in preliminary study, we have tried co-administration of several reagents those induce or mimic co-stimulation of antigen presenting cells by activated helper T cells. However, none of them including influenza virus derived helper T cell epitope (NP_311–325_, I-A^b^ restricted) and stimulatory anti-CD40 antibody did not show any improvement in both lethal rate and symptom progression after virus challenge at memory CTL phase in H5N1 virus *in vivo* protection assay (data not shown). We speculate that this invalidity of helper T cell help at memory CTL phase may be relying on property of A24Tg, virulence of H5N1 virus or immunization method using liposome peptide. In any case, further refinement of vaccination strategies may be required for their practical use. For example, annual i.n. vaccination may be required to recruit influenza antigen specific CTLs into the airway epithelia since the protection afforded by CTL peptide vaccines during the effector phase in the lung is more effective than that during the memory phase.

In conclusion, we have identified potent epitope peptides which can be used in a cross-protective CTL-inducing human vaccine against various influenza A virus subtypes using an *in vivo* HLA-A*2402 restricted epitope peptide selection system. It was observed that the efficacy of CTL inducing peptide vaccines against influenza A virus infection depended largely on two factors; whether the affinity/avidity of epitope peptides to the T cell receptor allows the stimulation of large viral antigen specific CTL populations, and the ability of these vaccines to recruit specific effector/memory CTLs in the lung. Therefore CTL peptide vaccines can be further modified to compensate for the shortcomings (such as virus subtype dependence, an inability of CTL induction etc.) of existing influenza vaccines and improve cross-protective responses against antigenic variants.

## Supporting Information

Figure S1 HLA-A*2402 restricted peptides do not induce epitope specific CTL in C57BL/6 mice. C57BL/6 mice were immunized s.c. twice with each HLA-A*2402 (human MHC class I) restricted peptide or H2D^b^ (C57BL/6 mice MHC class I) restricted peptide at 7 days interval. Seven days after the final immunization, bright CFSE-labeled target cells pulsed with immunized peptide and dim CFSE-labeled target cells pulsed with irrelevant peptide were injected i.v. as an *in vivo* CTL killing assay. Viability of the target cells in the spleen was examined at 20 h after injection. Epitope specific cell reduction ratios were calculated using the formula described in Materials and Methods.
(TIF)

Figure S2
Stabilization assay of the three highly immunogenic peptides to HLA-A*2402. The restriction of PA_130–138_, PB1_430–438_ or PB2_549–557_ peptides to HLA-A*2402 was examined by using RMA-S- A*2402 cells. Mean fluorescence intensity (MFI) was recorded at 1, 10 and 100 µM of peptide. The stability of HLA-A*2402 was evaluated by the delta percent mean fluorescence intensity (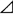
MFI %) increase of the HLA-A*2402 detected by staining with anti-HLA-A24 antibody.(TIF)

Figure S3 **Non-specific lung tissue disruption/inflammation by CpG-ODN administration is not observed.** A24Tg mice were immunized i.n. three times at 7 days interval with PBS alone or CpG-ODN plus empty-liposome solution. Lungs were harvested at day 7 after the final administration, preserved in 4% formalin, embedded in O.C.T. compound, frozen in dry ice-2-propanol, and 5 µm thick frozen sections were prepared. The sections were stained with Hematoxylin & Eosin.
(TIF)

File S1 **Supplementary Materials and Methods.**
(DOC)

## References

[1] de Jong JC, Palache AM, Beyer WE, Rimmelzwaan GF, Boon AC (2003). Haemagglutination-inhibiting antibody to influenza virus.. Dev Biol (Basel).

[2] Cox NJ, Subbarao K (1999). Influenza.. Lancet.

[3] Takada A, Shimizu Y, Kida H (1994). Protection of mice against Aujeszky's disease virus infection by intranasal vaccination with inactivated virus.. J Vet Med Sci.

[4] Takada A, Kida H (1996). Protective immune response of chickens against Newcastle disease, induced by the intranasal vaccination with inactivated virus.. Vet Microbiol.

[5] Ada GL, Jones PD (1986). The immune response to influenza infection.. Curr Top Microbiol Immunol.

[6] Fiore AE, Bridges CB, Cox NJ (2009). Seasonal influenza vaccines.. Curr Top Microbiol Immunol.

[7] Nichol KL (2008). Efficacy and effectiveness of influenza vaccination.. Vaccine.

[8] Alsharifi M, Furuya Y, Bowden TR, Lobigs M, Koskinen A (2009). Intranasal flu vaccine protective against seasonal and H5N1 avian influenza infections.. PLoS One.

[9] Bender BS, Croghan T, Zhang L, Small PA (1992). Transgenic mice lacking class I major histocompatibility complex-restricted T cells have delayed viral clearance and increased mortality after influenza virus challenge.. J Exp Med.

[10] McMichael AJ, Gotch FM, Noble GR, Beare PA (1983). Cytotoxic T-cell immunity to influenza.. N Engl J Med.

[11] Zweerink HJ, Courtneidge SA, Skehel JJ, Crumpton MJ, Askonas BA (1977). Cytotoxic T cells kill influenza virus infected cells but do not distinguish between serologically distinct type A viruses.. Nature.

[12] Kees U, Krammer PH (1984). Most influenza A virus-specific memory cytotoxic T lymphocytes react with antigenic epitopes associated with internal virus determinants.. J Exp Med.

[13] Townsend AR, Skehel JJ (1984). The influenza A virus nucleoprotein gene controls the induction of both subtype specific and cross-reactive cytotoxic T cells.. J Exp Med.

[14] Gotch F, McMichael A, Smith G, Moss B (1987). Identification of viral molecules recognized by influenza-specific human cytotoxic T lymphocytes.. J Exp Med.

[15] Yap KL, Ada GL, McKenzie IF (1978). Transfer of specific cytotoxic T lymphocytes protects mice inoculated with influenza virus.. Nature.

[16] Yap KL, Ada GL (1978). The recovery of mice from influenza virus infection: adoptive transfer of immunity with immune T lymphocytes.. Scand J Immunol.

[17] Wells MA, Ennis FA, Albrecht P (1981). Recovery from a viral respiratory infection. II. Passive transfer of immune spleen cells to mice with influenza pneumonia.. J Immunol.

[18] Lukacher AE, Braciale VL, Braciale TJ (1984). In vivo effector function of influenza virus-specific cytotoxic T lymphocyte clones is highly specific.. J Exp Med.

[19] Liang S, Mozdzanowska K, Palladino G, Gerhard W (1994). Heterosubtypic immunity to influenza type A virus in mice. Effector mechanisms and their longevity.. J Immunol.

[20] Seo SH, Peiris M, Webster RG (2002). Protective cross-reactive cellular immunity to lethal A/Goose/Guangdong/1/96-like H5N1 influenza virus is correlated with the proportion of pulmonary CD8(+) T cells expressing gamma interferon.. J Virol.

[21] Seo SH, Webster RG (2001). Cross-reactive, cell-mediated immunity and protection of chickens from lethal H5N1 influenza virus infection in Hong Kong poultry markets.. J Virol.

[22] Kreijtz JH, Bodewes R, van Amerongen G, Kuiken T, Fouchier RA (2007). Primary influenza A virus infection induces cross-protective immunity against a lethal infection with a heterosubtypic virus strain in mice.. Vaccine.

[23] Purcell AW, McCluskey J, Rossjohn J (2007). More than one reason to rethink the use of peptides in vaccine design.. Nat Rev Drug Discov.

[24] Buus S, Lauemoller SL, Worning P, Kesmir C, Frimurer T (2003). Sensitive quantitative predictions of peptide-MHC binding by a ‘Query by Committee’ artificial neural network approach.. Tissue Antigens.

[25] Nielsen M, Lundegaard C, Worning P, Lauemoller SL, Lamberth K (2003). Reliable prediction of T-cell epitopes using neural networks with novel sequence representations.. Protein Sci.

[26] Nielsen M, Lundegaard C, Worning P, Hvid CS, Lamberth K (2004). Improved prediction of MHC class I and class II epitopes using a novel Gibbs sampling approach.. Bioinformatics.

[27] Larsen MV, Lundegaard C, Lamberth K, Buus S, Brunak S (2005). An integrative approach to CTL epitope prediction: a combined algorithm integrating MHC class I binding, TAP transport efficiency, and proteasomal cleavage predictions.. Eur J Immunol.

[28] Sette A, Sidney J (1999). Nine major HLA class I supertypes account for the vast preponderance of HLA-A and -B polymorphism.. Immunogenetics.

[29] Nagata T, Toyota T, Ishigaki H, Ichihashi T, Kajino K (2007). Peptides coupled to the surface of a kind of liposome protect infection of influenza viruses.. Vaccine.

[30] Taneichi M, Ishida H, Kajino K, Ogasawara K, Tanaka Y (2006). Antigen chemically coupled to the surface of liposomes are cross-presented to CD8+ T cells and induce potent antitumor immunity.. J Immunol.

[31] Takada A, Matsushita S, Ninomiya A, Kawaoka Y, Kida H (2003). Intranasal immunization with formalin-inactivated virus vaccine induces a broad spectrum of heterosubtypic immunity against influenza A virus infection in mice.. Vaccine.

[32] Nakano Y, Mori M, Nishinohara S, Takita Y, Naito S (2001). Surface-linked liposomal antigen induces IgE-selective unresponsiveness regardless of the lipid components of liposomes.. Bioconjug Chem.

[33] Nakano Y, Mori M, Nishinohara S, Takita Y, Naito S (1999). Antigen-specific, IgE-selective unresponsiveness induced by antigen-liposome conjugates. Comparison of four different conjugation methods for the coupling of antigen to liposome.. Int Arch Allergy Immunol.

[34] Naruse H, Ogasawara K, Kaneda R, Hatakeyama S, Itoh T (1994). A potential peptide vaccine against two different strains of influenza virus isolated at intervals of about 10 years.. Proc Natl Acad Sci U S A.

[35] Belyakov IM, Ahlers JD, Brandwein BY, Earl P, Kelsall BL (1998). The importance of local mucosal HIV-specific CD8(+) cytotoxic T lymphocytes for resistance to mucosal viral transmission in mice and enhancement of resistance by local administration of IL-12.. J Clin Invest.

[36] Kos FJ, Mullbacher A (1992). Enhancement of antigen-specific activation of CD8+ memory cytotoxic T cells by B cell-derived factors.. Immunobiology.

[37] Liu Y, Mullbacher A (1989). Activated B cells can deliver help for the in vitro generation of antiviral cytotoxic T cells.. Proc Natl Acad Sci U S A.

[38] Thomas PG, Keating R, Hulse-Post DJ, Doherty PC (2006). Cell-mediated protection in influenza infection.. Emerg Infect Dis.

[39] Wang F, Bade E, Kuniyoshi C, Spears L, Jeffery G (1999). Phase I trial of a MART-1 peptide vaccine with incomplete Freund's adjuvant for resected high-risk melanoma.. Clin Cancer Res.

[40] Gohara R, Imai N, Rikimaru T, Yamada A, Hida N (2002). Phase 1 clinical study of cyclophilin B peptide vaccine for patients with lung cancer.. J Immunother.

[41] Doherty PC, Allan W, Boyle DB, Coupar BE, Andrew ME (1989). Recombinant vaccinia viruses and the development of immunization strategies using influenza virus.. J Infect Dis.

[42] Lin YL, Askonas BA (1981). Biological properties of an influenza A virus-specific killer T cell clone. Inhibition of virus replication in vivo and induction of delayed-type hypersensitivity reactions.. J Exp Med.

[43] Flynn KJ, Belz GT, Altman JD, Ahmed R, Woodland DL (1998). Virus-specific CD8+ T cells in primary and secondary influenza pneumonia.. Immunity.

[44] O'Neill E, Krauss SL, Riberdy JM, Webster RG, Woodland DL (2000). Heterologous protection against lethal A/HongKong/156/97 (H5N1) influenza virus infection in C57BL/6 mice.. J Gen Virol.

[45] Kreijtz JH, de Mutsert G, van Baalen CA, Fouchier RA, Osterhaus AD (2008). Cross-recognition of avian H5N1 influenza virus by human cytotoxic T-lymphocyte populations directed to human influenza A virus.. J Virol.

[46] La Gruta NL, Kedzierska K, Pang K, Webby R, Davenport M (2006). A virus-specific CD8+ T cell immunodominance hierarchy determined by antigen dose and precursor frequencies.. Proc Natl Acad Sci U S A.

[47] Ely KH, Cookenham T, Roberts AD, Woodland DL (2006). Memory T cellpopulations in the lung airways are maintained by continual recruitment.. J Immunol.

[48] Day EB, Zeng W, Doherty PC, Jackson DC, Kedzierska K (2007). The context of epitope presentation can influence functional quality of recalled influenza A virus-specific memory CD8+ T cells.. J Immunol United States.

[49] Deliyannis G, Jackson DC, Ede NJ, Zeng W, Hourdakis I (2002). Induction of long-term memory CD8(+) T cells for recall of viral clearing responses against influenza virus.. J Virol.

[50] Deliyannis G, Kedzierska K, Lau YF, Zeng W, Turner SJ (2006). Intranasal lipopeptide primes lung-resident memory CD8+ T cells for long-term pulmonary protection against influenza.. Eur J Immunol.

