# Supplementary figures and images for: Cross-Protective Peptide Vaccine against Influenza A Viruses Developed in HLA-A*2402 Human Immunity Model
Source: PLoS One. 2011 Sep 19;6(9):e24626. doi: 10.1371/journal.pone.0024626 (PMC3176274; doi:10.1371/journal.pone.0024626)

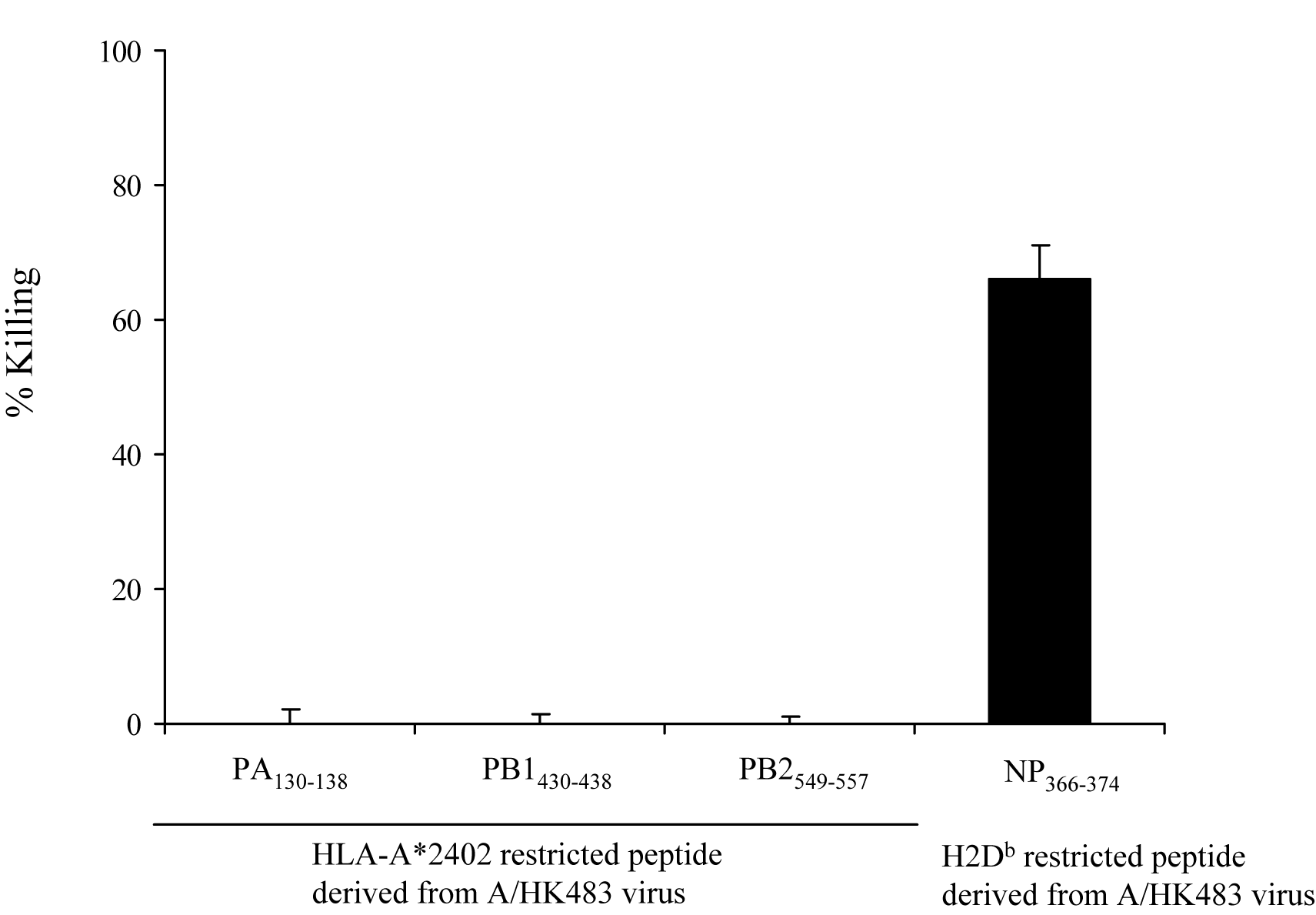

Supplement: Figure S1 — HLA-A*2402 restricted peptides do not induce epitope specific CTL in C57BL/6 mice. C57BL/6 mice were immunized s.c. twice with each HLA-A*2402 (human MHC class I) restricted peptide or H2Db (C57BL/6 mice MHC class I) restricted peptide at 7 days interval. Seven days after the final immunization, bright CFSE-labeled target cells pulsed with immunized peptide and dim CFSE-labeled target cells pulsed with irrelevant peptide were injected i.v. as an in vivo CTL killing assay. Viability of the target cells in the spleen was examined at 20 h after injection. Epitope specific cell reduction ratios were calculated using the formula described in Materials and Methods. (TIF) [file pone.0024626.s001.tif]

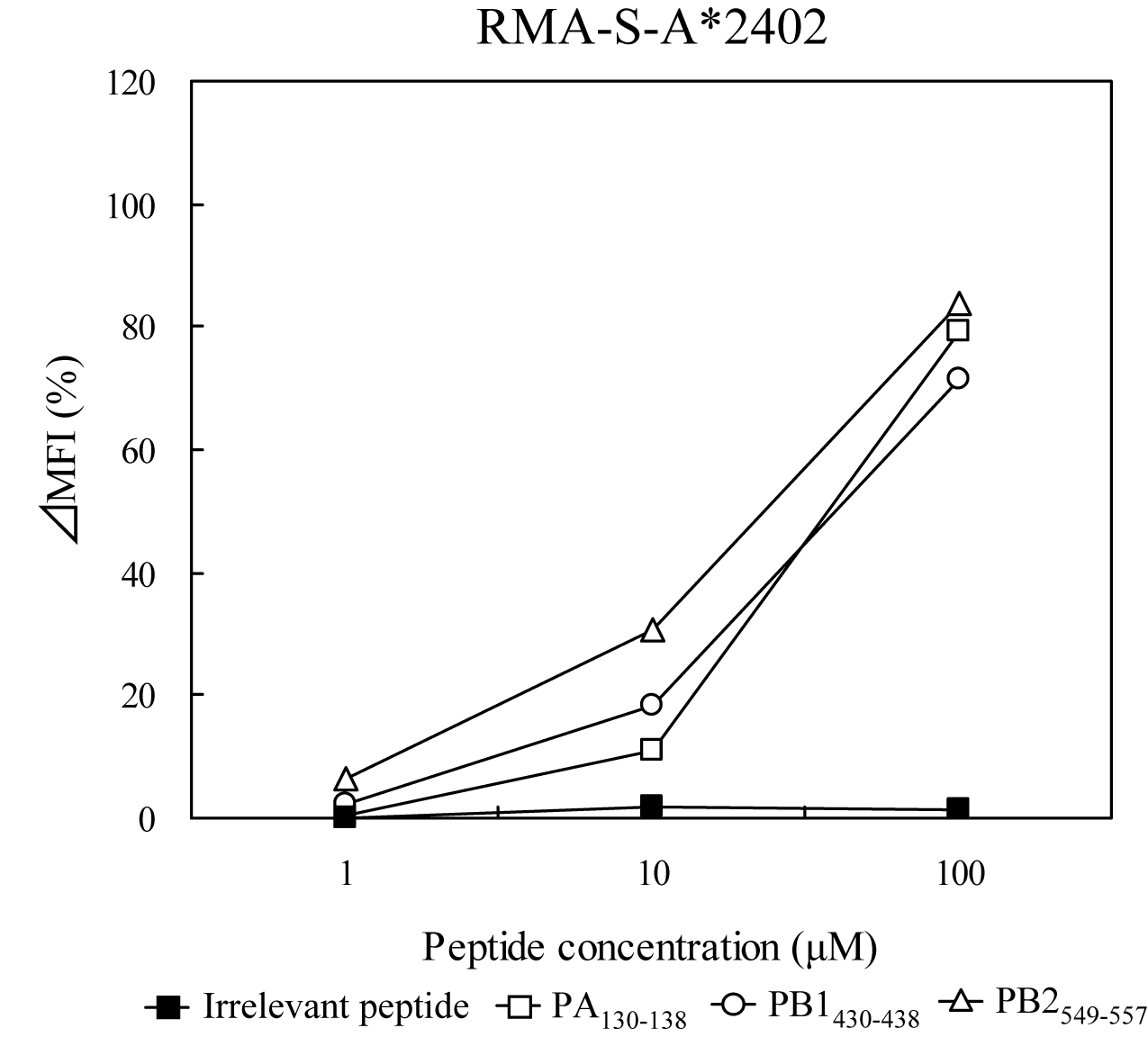

Supplement: Figure S2 — Stabilization assay of the three highly immunogenic peptides to HLA-A*2402. The restriction of PA130–138, PB1430–438 or PB2549–557 peptides to HLA-A*2402 was examined by using RMA-S- A*2402 cells. Mean fluorescence intensity (MFI) was recorded at 1, 10 and 100 µM of peptide. The stability of HLA-A*2402 was evaluated by the delta percent mean fluorescence intensity (MFI %) increase of the HLA-A*2402 detected by staining with anti-HLA-A24 antibody. (TIF) [file pone.0024626.s002.tif]

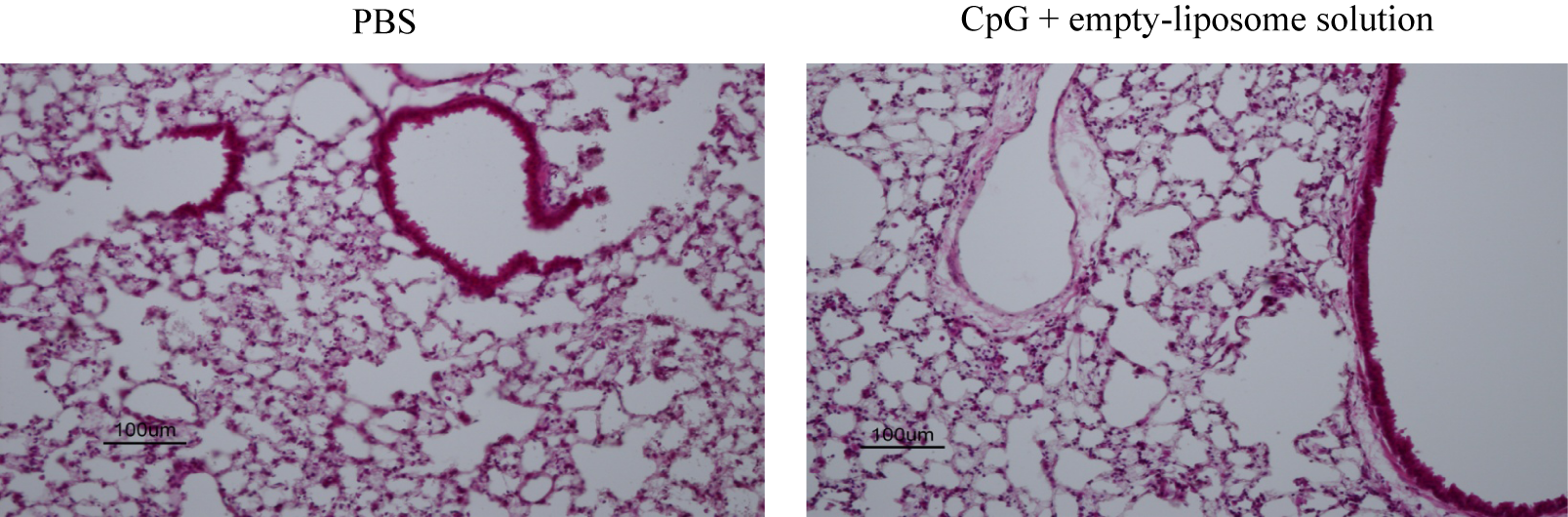

Supplement: Figure S3 — Non-specific lung tissue disruption/inflammation by CpG-ODN administration is not observed. A24Tg mice were immunized i.n. three times at 7 days interval with PBS alone or CpG-ODN plus empty-liposome solution. Lungs were harvested at day 7 after the final administration, preserved in 4% formalin, embedded in O.C.T. compound, frozen in dry ice-2-propanol, and 5 µm thick frozen sections were prepared. The sections were stained with Hematoxylin & Eosin. (TIF) [file pone.0024626.s003.tif]
