# Supplementary material for: Cross-Protective Peptide Vaccine against Influenza A Viruses Developed in HLA-A*2402 Human Immunity Model
Source: PLoS One. 2011 Sep 19;6(9):e24626. doi: 10.1371/journal.pone.0024626 (PMC3176274; doi:10.1371/journal.pone.0024626)
Supplement: File S1 — Supplementary Materials and Methods. (DOC) [file pone.0024626.s004.doc]

**Supplementary Materials and Methods**

**Mice**

C57BL/6 mice (6 weeks old) obtained from Japan SLC, Inc. (Hamamatsu, Japan) were bread under specific-pathogen-free conditions. All experimental procedures were approved by Hokkaido University Animal Care and Use Committee, Sapporo, Japan (approval number 10-0060).

***In vivo* cytotoxicity assays**

Eight to 12 week-old C57BL/6 mice were immunized s.c. twice with each liposome-conjugated peptide in the presence of CpG-ODN (CpG 5002, 5 μg/mouse) at 7 days interval. Splenocytes from C57BL/6 mice were suspended in PBS and then labeled with two different concentrations (5 μM or 0.5 μM) of CFDA-SE at room temperature for 10 min. After addition of equal volumes of heat inactivated rabbit serum to quench the CFSE labeling reaction, cells were washed twice with PBS. Cells were further incubated with 0.5 μM immunizing peptide or an irrelevant peptide for 2 h at 37℃ and 5% CO_2_. Five million cells cultured with respective peptides were mixed together and inoculated i.v. into immunized mice. Twenty hours after target cells were inoculated, splenocytes were harvested and CFSE-positive cells were analyzed by flow cytometry with dead cell exclusion performed by propidium iodide staining. Peptide specific cell reduction ratios were calculated using the formula described in Materials and Methods.

**Cell lines**

TAP-2-deficient mouse cell line RMA-S cells were deficient in antigen processing.　They expressed MHC class I molecules at the cell surface, especially at reduced temperatures (26°C). The HLA-A*2402 gene was transfected into RMA-S cells by using HIV-based lentiviral vector. Stably transfected cells were established in the presence of puromycin (50 μg/ml).

**HLA stabilization assay**

The restriction of the peptides to HLA-A*2402 was examined by using HLA stabilization assay. RMA-S- A*2402 cells (2 ×10^5^ cells per 5 ml polypropylene tube) were cultured at 26℃ for 18 h in 100 μl RPMI 1640 (Invitrogen) supplemented with 10% fetal bovine serum (FBS) followed by incubation with 100 μl RPMI 1640 containing 1-100 μM peptides and human β2 microglobulin (Acris Antibodies, San Diego, CA) (5 μg/mL) at 26℃ for 2 h and then at 37℃ for 2 h. M1_58-66_ peptide (HLA-A*0201 restricted peptide derived from influenza A virus M1 protein) was used as an irrelevant peptide. The cells were washed with PBS and then incubated for 30 min on ice with a PE-conjugated anti-HLA-A24 antibody (MBL). Dead cells were labeled with 7-aminoactinomycinD (Invitrogen). Stained cells were quantified using a FACSCant flow cytometer (Becton Dickinson). The stability of HLA-A*2402 was evaluated by the delta percent mean fluorescence intensity (⊿ MFI %) increase of the HLA-A*2402 detected by staining with anti-HLA-A24 antibody : ⊿ MFI % = [( MFI induced by 1-100 μM peptide at 37℃－MFI induced by PBS at 37℃) / ( MFI induced by 100 μM peptide at 26℃－MFI induced by PBS at 37℃)] ×100%.

**Histological analysis of lung tissues**

Eight to 12 week-old A24Tg mice were immunized i.n. three times weekly interval with PBS alone or CpG-ODN plus empty-liposome solution. Seven days after the final immunization, lungs were harvested, preserved in 4% formalin, embedded in O.C.T. (Optimal Cutting Temperature) compound (Sakura) and slowly frozen in dry ice-2-propanol. Five μm thick frozen sections were prepared in a cryostat and air-dried for 1 hour at room temperature. The sections were rehydrated in PBS and stained with Hematoxylin & Eosin.
